# Nutrient Solution Flowing Environment Affects Metabolite Synthesis Inducing Root Thigmomorphogenesis of Lettuce (*Lactuca sativa* L.) in Hydroponics

**DOI:** 10.3390/ijms242316616

**Published:** 2023-11-22

**Authors:** Bateer Baiyin, Yue Xiang, Jiangtao Hu, Kotaro Tagawa, Jung Eek Son, Satoshi Yamada, Qichang Yang

**Affiliations:** 1 Research Center for Smart Horticulture Engineering, Chengdu National Agricultural Science & Technology Center, Institute of Urban Agriculture, Chinese Academy of Agricultural Sciences, Chengdu 610213, China; bater@caas.cn (B.B.); xiangyue@caas.cn (Y.X.); hujiangtao@caas.cn (J.H.); 2 Faculty of Agriculture, Tottori University, Tottori 680-8553, Japan; tagawa@tottori-u.ac.jp (K.T.); syamada@tottori-u.ac.jp (S.Y.); 3 Department of Agriculture, Forestry and Bioresources, Seoul National University, Seoul 08826, Republic of Korea; sjeenv@snu.ac.kr

**Keywords:** thigmomorphogenesis, nutrient solution flow, mechanical stimulation, root morphology, hydroponics, metabolomics

## Abstract

The principal difference between hydroponics and other substrate cultivation methods is the flowing liquid hydroponic cultivation substrate. Our previous studies have revealed that a suitable flowing environment of nutrient solution promoted root development and plant growth, while an excess flow environment was unfavorable for plants. To explain the thigmomorphogenetic response of excess flow-induced metabolic changes, six groups of lettuce (*Lactuca sativa* L.), including two flow conditions and three time periods, were grown. Compared with the plants without flow, the plants with flow showed decreased root fresh weight, total root length, root surface area, and root volume but increased average root diameter and root density. The roots with flow had more upregulated metabolites than those without flow, suggesting that the flow may trigger metabolic synthesis and activity. Seventy-nine common differential metabolites among six groups were screened, and enrichment analysis showed the most significant enrichment in the arginine biosynthesis pathway. Arginine was present in all the groups and exhibited greater concentrations in roots with flow than without flow. It can be speculated from the results that a high-flowing environment of nutrient solution promotes arginine synthesis, resulting in changes in root morphology. The findings provide insights on root thigmomorphogenesis affected by its growing conditions and help understand how plants respond to environmental mechanical forces.

## 1. Introduction

Hydroponics uses nutrient solutions to provide plants with the necessary nutrients and a suitable environment for growth, thereby eliminating the need for soil. This cultivation method can save land and allow for the precise control of nutrients and water provided to plants [1]. Compared with soil cultures, hydroponics provides a more stable growing environment, thus allowing plants to grow faster and produce higher yields while reducing the spread of soil-borne diseases and pests [2]. Furthermore, hydroponics plays an important role in special settings, such as vertical farms and urban agriculture. Despite some technical challenges, hydroponics has been widely applied in modern agriculture and has great development potential [3].

The main difference between hydroponics and soil- or substrate-based cultivation is that hydroponics uses a flowing medium, like nutrient solution, as the cultivation substrate. This nutrient solution offers several unique advantages [4]. First, it promotes the circulation and even distribution of nutrient ions within the cultivation system and can deliver nutrient ions to plant roots via the circulation system, thereby preventing the formation of nutrient-depleted zones and ensuring an adequate nutrient supply to roots. Adequate nutrient delivery is one of the reasons for rapid plant growth [5]. Second, the flow of the nutrient solution provides mechanical stimulation to the roots [6], exerting pressure and friction on the plant roots that induce root thigmomorphogenesis.

Thigmomorphogenesis [7] is a phenomenon in which mechanical stimulation influences plant growth, morphology, and development. Plants adjust and establish their own morphological structures in response to physical interactions with their surrounding environment [8,9]. This mechanism allows plants to adaptively respond to external stimuli and environmental conditions. During thigmomorphogenesis, plants perceive and respond to mechanical stimuli by regulating their growth direction and morphology to construct specific tissue structures [10]. This allows plants to establish suitable morphological structures under different environmental conditions to maximize their adaptations to and survival in their current environment. Typically, plants undergo significant morphological changes, such as dwarfing, elongation, stem mechanical property alterations, delayed flowering, increased root anchorage strength, and reduced stomatal aperture [10,11,12].

The root is a vital organ for nutrient absorption in plants, and its morphology and structure significantly affect nutrient uptake [13]. Thigmomorphogenesis occurs when roots experience pressure and friction from the flow of a nutrient solution. Roots adjust their morphology to adapt to the specific flow-field conditions of the nutrient solution, which directly affects the nutrient absorption capacity of the plant and indirectly influences plant growth and yield [6,14]. Several studies have been conducted on plant thigmomorphogenesis, with most focusing on the morphological adjustments and establishment of aboveground plant parts in response to wind [15], rain [16], hail [17], animal touch [18], and other mechanical forces. These studies have mainly examined morphological changes in the stem, leaf, and flower, such as the leaning response of trees and long-term deformation of stems [19,20,21] induced by aerial environmental factors. This could be attributed to the ease of observing and studying aerial environments and the resulting morphological changes, as well as their greater relevance and attention received in the fields of biology and agriculture. However, few studies have investigated thigmomorphogenesis in plant roots. The scarcity of research in this field may be due to various reasons, including technical difficulties in controlling rootzone environments, observing and measuring root morphological changes induced by underground environmental factors, and a relatively limited understanding of the rootzone environment of plants. In particular, research on root thigmomorphogenesis induced by nutrient solution flow is limited.

Thigmomorphogenesis is now attracting great interest from researchers. Studies have revealed the mechanisms of thigmomorphogenesis from the perspectives of hormone, epigenetic modification, and gene regulation. For instance, GA_4_ was reduced by touch in *Arobidopsis*, inhibiting its growth [22]. The phosphorylation of TREPH1 induced by touch resulted in delayed bolting [23]. Touch activated ethylene signaling, and *PGX3* was inhibited by EIN3, which reduced cell elongation [24].

Plant development is determined by both genetic and environmental factors, and metabolites play a key role in the process of phenotype formation [25,26]. Under the flow of a nutrient solution, metabolites are closely connected with the perception and response of roots to flow stimuli. These metabolites regulate and transmit signals within plants that can influence their physiological responses and morphological changes in response to environmental stimuli [27]. Furthermore, the composition and content of metabolites vary with plant growth stage. Plants produce different types and amounts of metabolites at different growth stages. These differences may involve changes in multiple metabolic pathways and key enzymatic activities, resulting in altered metabolite synthesis and regulation [28]. When analyzing the mechanism of thigmomorphogenesis, it is necessary to consider the differences in metabolites at different growth stages. Understanding the role of different metabolites in plants at different growth stages in the process of thigmomorphogenesis caused by nutrient solution flow is crucial for a better understanding of the mechanisms of adaptive responses and morphological adjustments in plant root systems.

Our previous studies have revealed that plant growth was promoted by a flow rate of no more than 4 L/min [5,6]. It is curious that a higher flow rate might allow for an adequate nutrient mixture and bring more oxygen; however, plant growth was inhibited. Therefore, this study was carried out by comparing two nutrient solution flow rates (S: 0 L/min, F: 30 L/min) for three sampling periods (1–3 weeks), resulting in a total of six treatments. The F1–F3 indicate sampling periods of 1–3 weeks, respectively, with a flow rate of 30 L/min, while S1–S3 indicate sampling periods of 1, 2, and 3 weeks, respectively, with a flow rate of 0 L/min. The results would elucidate the thigmomorphogenetic response of lettuce root under a high-flowing environment of nutrient solution. By comparing the composition and content of root metabolites between the two conditions, potential metabolic pathways associated with the response to flowing stimuli were identified.

## 2. Results

### 2.1. Effects of High-Flowing Environment on Plant Growth and Root Morphology with Growth Stage

**Figure 1:**
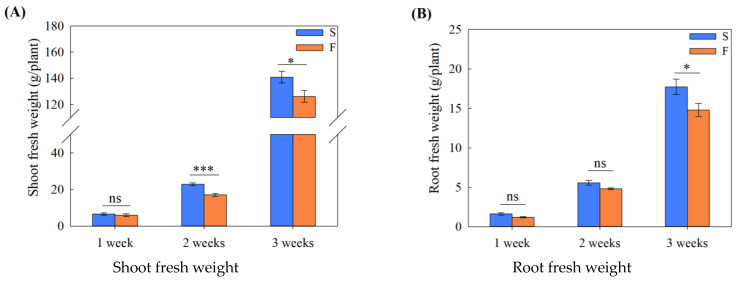

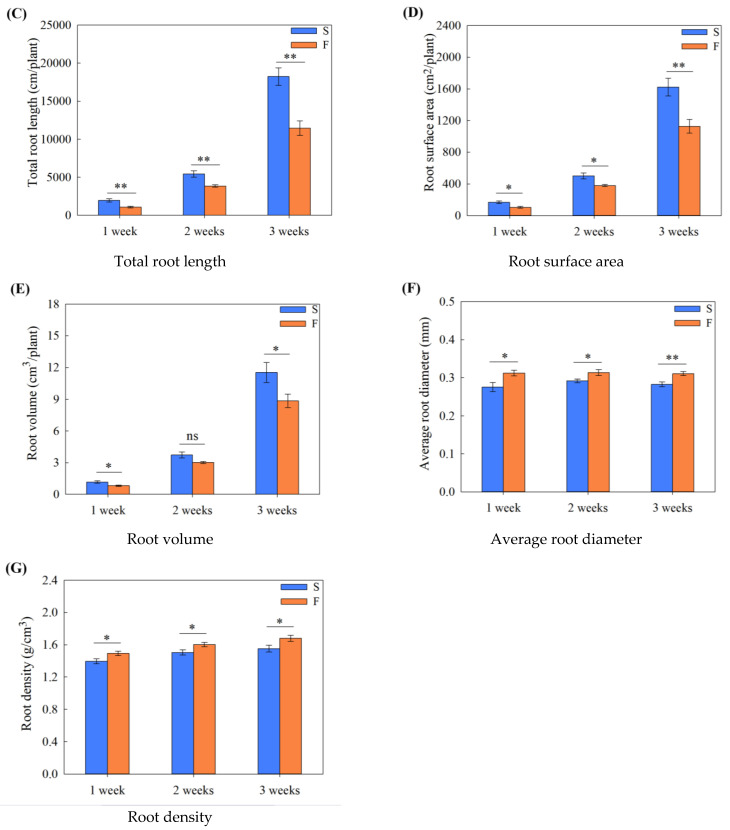
Root (**A**) and shoot (**B**) fresh weights and root morphology, including length (**C**), surface area (**D**), volume (**E**), diameter (**F**), and density (**G**), under different treatments. All data were statistically analyzed using independent sample *t*-tests, and the statistical results were expressed as the mean ± standard error (*n* = 5). A value of *p* ≤ 0.05 indicated significant differences. *: *p* ≤ 0.05; **: *p* ≤ 0.01; ***: *p* ≤ 0.001; ”ns” means no significant difference.

Compared with S2 and S3, the shoot fresh weights of F2 and F3 plants were significantly decreased by 25.3% and 10.4%, respectively, whereas significant differences were not observed between S1 and F1 (Figure 1A). Only F3 and S3 showed significant differences in root fresh weight, and their change trend was similar to that of shoot fresh weight (Figure 1B). The flow of the nutrient solution had a significant effect on the root morphology at different sampling periods. Specifically, compared with S1–S3, the total root length of F1–F3 decreased by 45.2%, 29.0%, and 37.2%, respectively (Figure 1C), and the root surface area decreased by 37.8%, 24.3%, and 30.6%, respectively (Figure 1D). Compared with S1 and S3, the root volumes of F1 and F3 were significantly lower (Figure 1E). However, changes in average root diameter and root density showed opposite trends compared with length and surface area. Compared with S1–S3, the average root diameters of F1–F3 significantly increased by 13.3%, 7.4%, and 9.8%, respectively (Figure 1F), and the root density increased by 7.0%, 6.4%, and 8.3%, respectively (Figure 1G).

### 2.2. Widely-Targeted Metabolite Detection

A total of 2139 metabolites were detected, including 320 phenolic acids, 279 terpenoids, 272 flavonoids, 208 amino acids and their derivatives, 204 lipids, 200 alkaloids, 140 organic acids, 105 lignans and coumarins, 82 nucleotides and their derivatives, 21 quinones, six steroids, four tannins, and 298 others. In total, 2128, 2127, 2126, 2115, 2133, and 2120 metabolites were detected in the S1, S3, S3, and F1–F3 groups, respectively.

**Figure 2:**
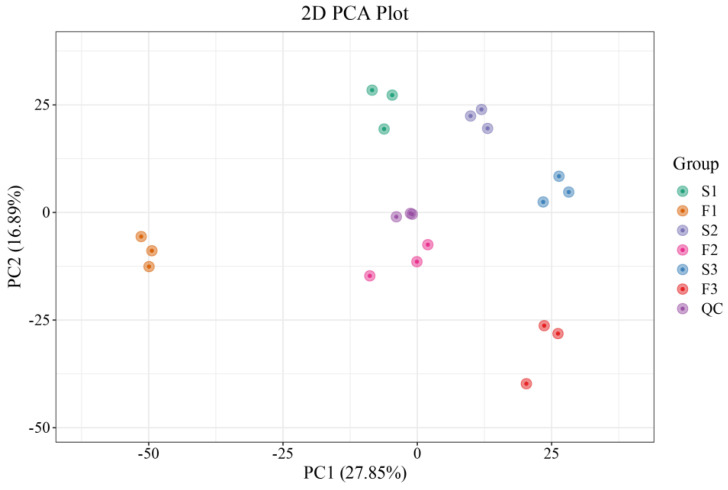
Result of PCA of roots under different treatments. F1–F3 indicate sampling periods of 1–3 weeks with a flow rate of 30 L/min, while S1–S3 indicate sampling periods of 1–3 weeks with a flow rate of 0 L/min, respectively.

In the principal component analysis (PCA) results, the six groups of samples showed significant separation, indicating significant changes in the metabolites in the treated samples (Figure 2). Simultaneously, the quality control (QC) samples clustered together and partially overlapped, indicating that the results of this study are reliable and repeatable. The first and second principal components explain 27.9% and 16.9% of the features in the original dataset, respectively.

**Figure 3:**
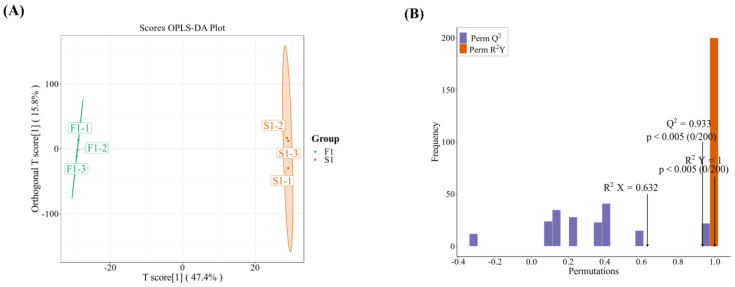

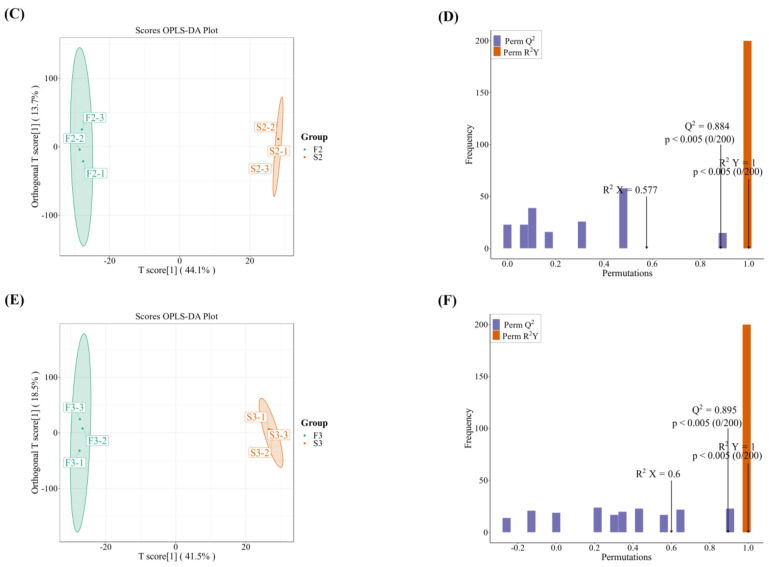
Result of OPLS-DA between F1 and S1 (**A**,**B**), F2 and S2 (**C**,**D**), and F3 and S3 (**E**,**F**). F1–F3 indicate sampling periods of 1–3 weeks with a flow rate of 30 L/min, while S1–S3 indicate sampling periods of 1–3 weeks with a flow rate of 0 L/min, respectively.

Orthogonal partial least squares discriminant analysis (OPLS-DA) analysis showed that R^2^X and R^2^Y represent the explanatory powers of the constructed model for the X and Y matrices, respectively. The Q^2^ of these three comparative groups was close to 0.9, indicating that the model was very good (Figure 3). The OPLS-DA score chart indicates a significant separation between the F and S treatments in the group comparisons for the three time periods.

**Figure 4:**
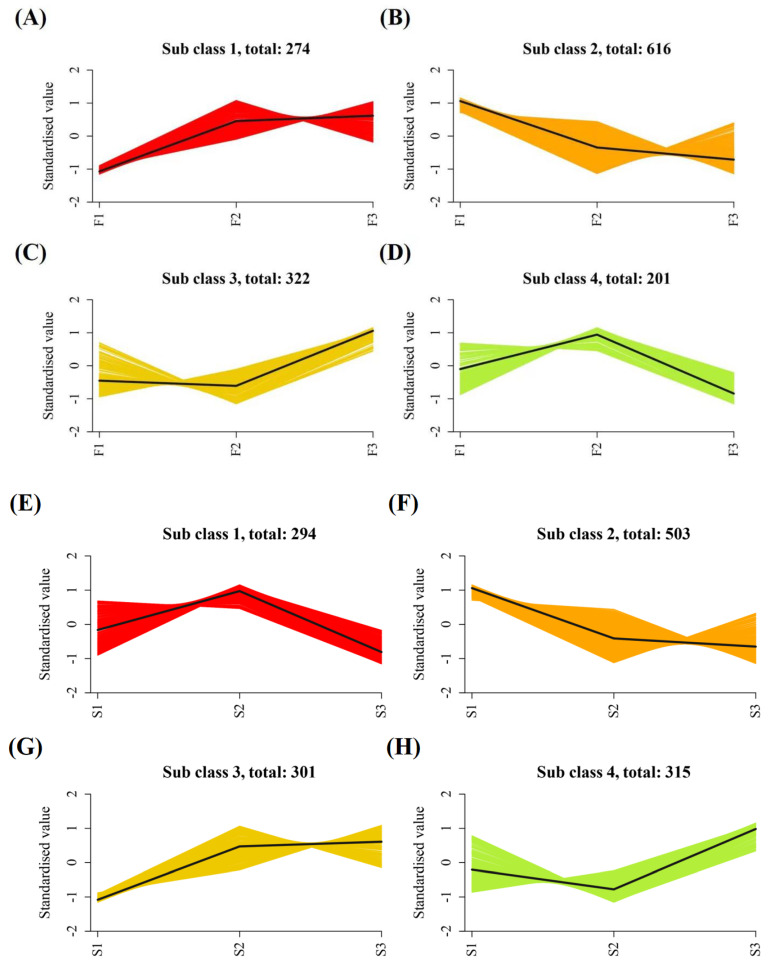
K—means maps of different metabolites under F (**A**–**D**) and S (**E**–**H**) treatments.

The K-means clustering analysis was conducted on the differential metabolites in all group comparisons. As shown in Figure 4A–D, in F1–F3, over time, 596 metabolites showed an upward trend, 616 showed a downward trend, and 201 metabolites showed an upward trend and then a downward trend. In S1–S3, over time, 616 metabolites showed an upward trend, 503 metabolites showed a downward trend, and the remaining 294 metabolites showed a trend of initially increasing and then decreasing (Figure 4E–H).

**Figure 5:**
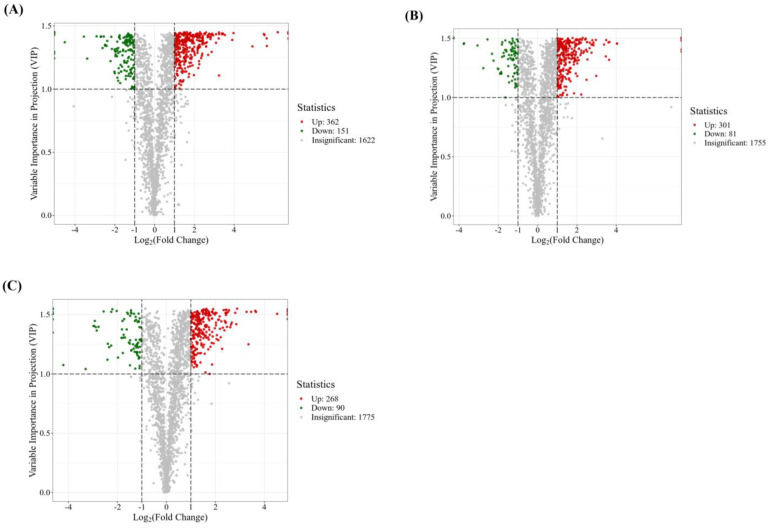
Volcano maps of different metabolites between F1 and S1 (**A**), F2 and S2 (**B**), and F3 and S3 (**C**). F1–F3 indicate sampling periods of 1–3 weeks with a flow rate of 30 L/min, while S1–S3 indicate sampling periods of 1–3 weeks with a flow rate of 0 L/min, respectively.

There were 513 differential metabolites identified in the comparison between the F1 and S1 groups, with 362 upregulated and 151 downregulated (Figure 5A); 382 differential metabolites were identified in the comparison between the F2 and S2 groups, with 301 upregulated and 81 downregulated (Figure 5B); and 358 differential metabolites were detected in the comparison between the F3 and S3 groups, with 268 upregulated and 90 downregulated (Figure 5C). In these three group comparisons, the number of upregulated metabolites was higher than that of downregulated metabolites, indicating that the nutrient solution flow may activate physiological and metabolic activities related to root morphological changes.

### 2.3. Key Differential Metabolites and Metabolic Pathways

**Figure 6:**
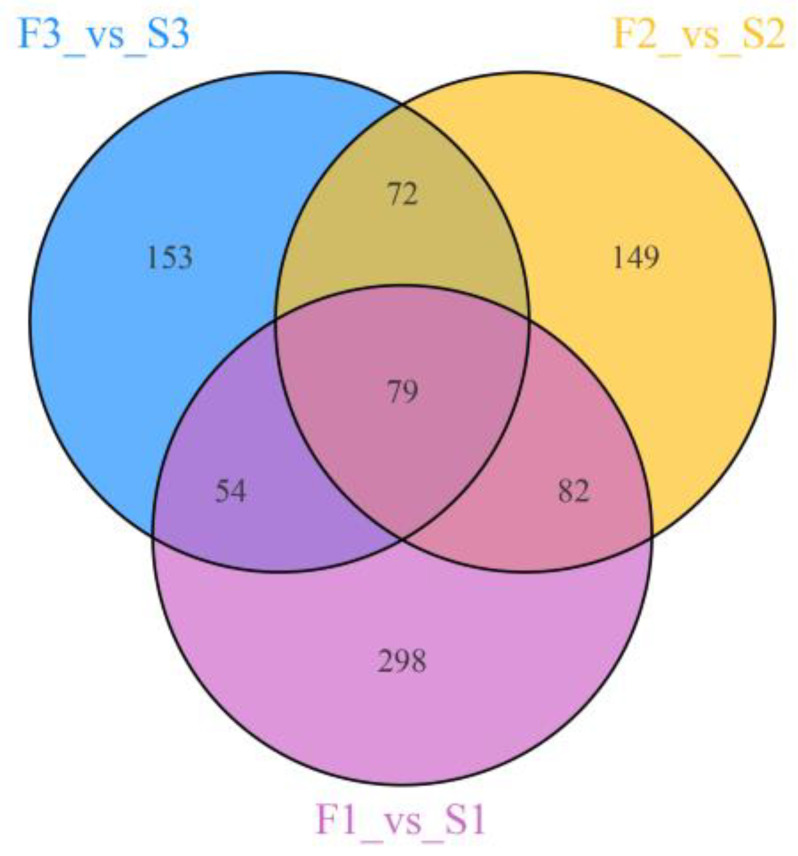
Wayne diagram of different metabolites under different treatments. F1–F3 indicate sampling periods of 1–3 weeks with a flow rate of 30 L/min, while S1–S3 indicate sampling periods of 1–3 weeks with a flow rate of 0 L/min, respectively.

The unique and common differential metabolites between F1 and S1, F2 and S2, and F3 and S3 are described using Wayne plots (Figure 6). Among these three comparative groups, 79 overlapping differential metabolites were considered key metabolites that affect root morphology.

**Figure 7:**
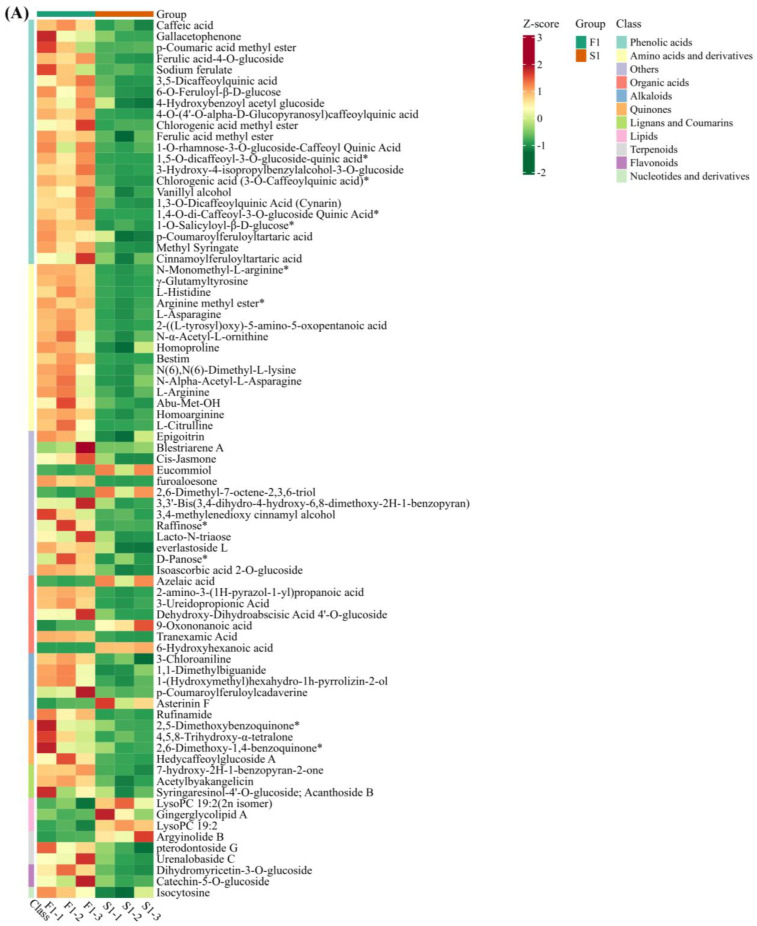

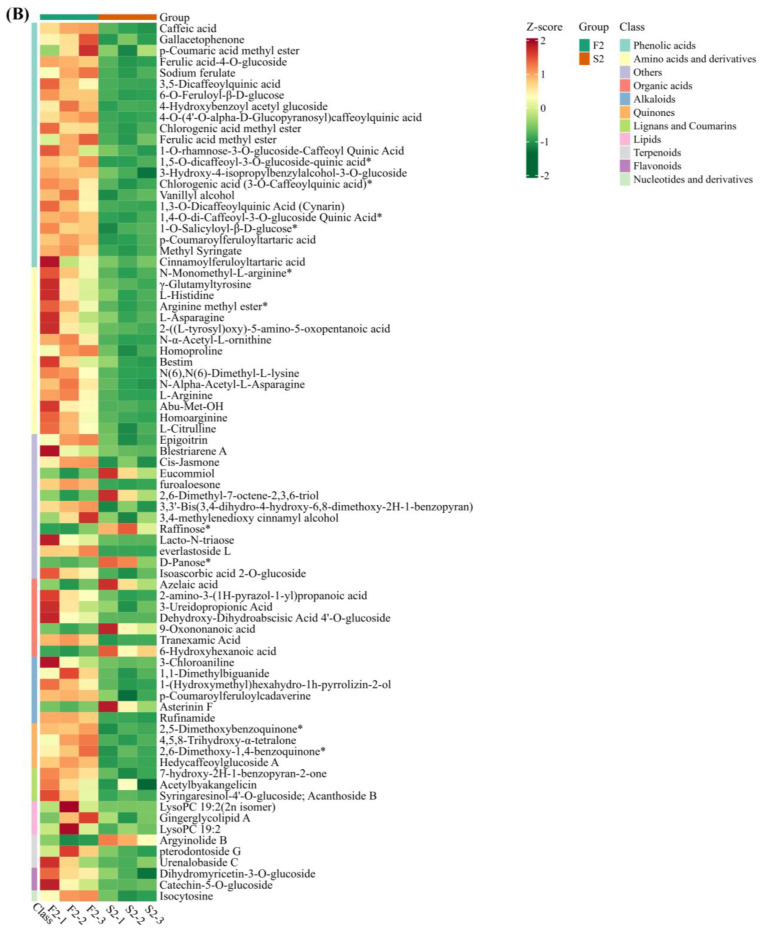

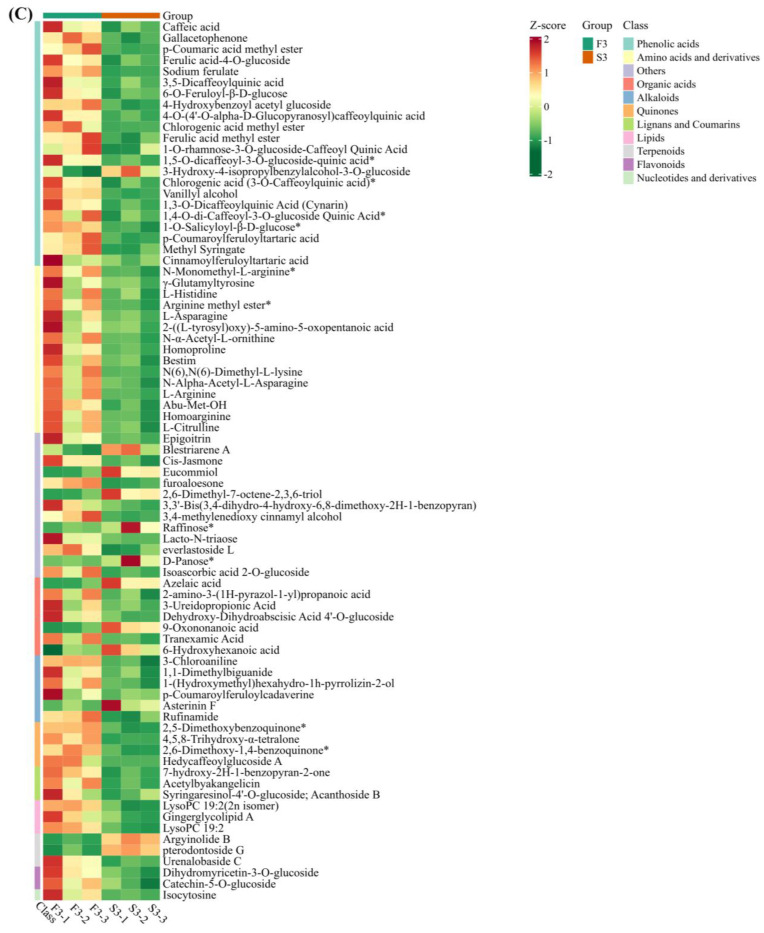
Cluster heat maps of common different metabolites between F1 and S1 (**A**), F2 and S2 (**B**), and F3 and S3 (**C**). F1–F3 indicate sampling periods of 1–3 weeks with a flow rate of 30 L/min, while S1–S3 indicate sampling periods of 1–3 weeks with a flow rate of 0 L/min, respectively, “*” means there are isomers.

**Figure 8:**
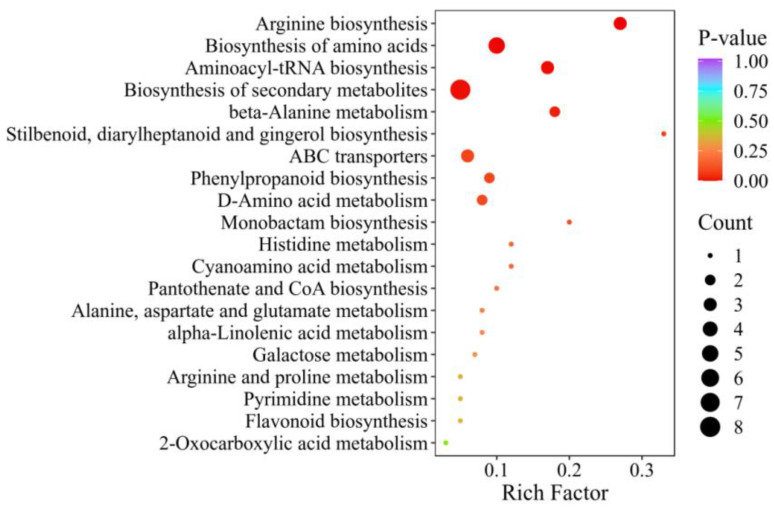
KEGG enrichment analysis of common differential metabolites.

A cluster analysis of these 79 key differential metabolites revealed that a total of 22 phenolic acids (including caffeic acid, gallic acetophenone, methyl p-coumaric acid, ferulic acid 4-O-glucoside, and sodium ferulate) and 15 amino acids and their derivatives (including L-histidine, L-asparagine, L-arginine, N-α-Acetyl-L-ornithine, and L-citrulline) were found to have a higher content in group F treatments (Figure 7). In addition, KEGG enrichment analysis was conducted on these 79 substances, which were mainly annotated and enriched in the arginine, amino acid, aminoacyl-tRNA, and secondary metabolite biosynthesis pathways (Figure 8).

**Figure 9:**
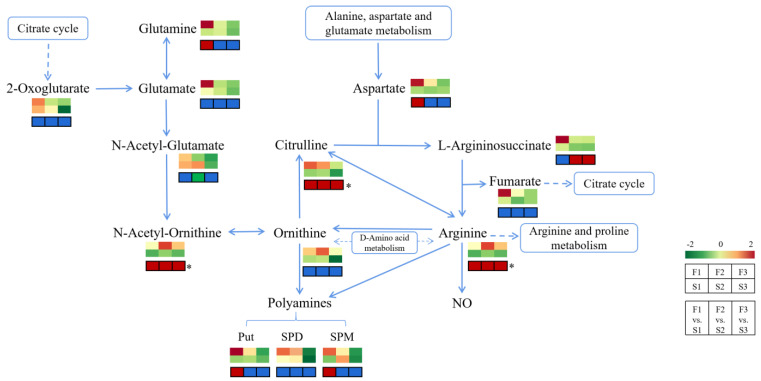
Changes of metabolites in the arginine biosynthesis pathway regulated by nutrient solution flow. Note: the top color block represents the relative content of metabolites in the six treatments, with red and green representing high and low content, respectively; the lower color block represents the up- or down-regulation of metabolites in the three comparison groups; red, blue, and green indicate that the metabolites were significantly up-regulated, not changed significantly, and significantly down-regulated, respectively. The “*” indicates that the metabolite belongs to 79 common differential metabolites.

**Figure 10:**
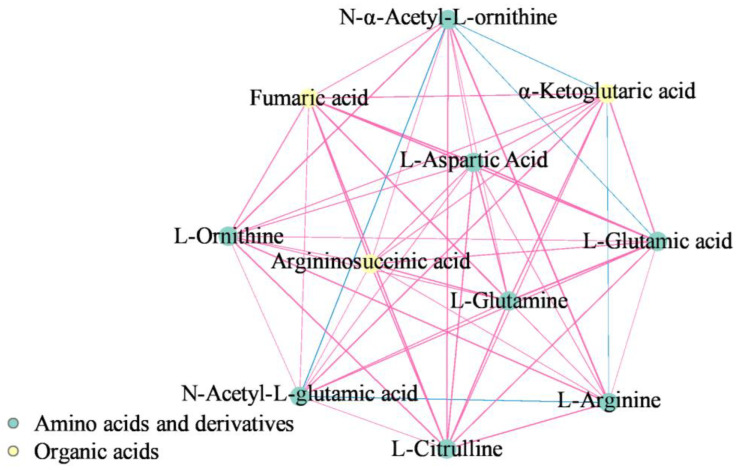
Network diagram of the interrelationships between metabolites in the arginine biosynthesis pathway. Note: the red line represents a positive correlation, and the blue line represents a negative correlation. The thickness of the line represents the absolute value of the Pearson correlation coefficient r. The thicker the line, the larger |r|.

The secondary metabolic biosynthesis pathways contained the most common differential metabolites (eight substances), followed by the amino acid (five substances), aminoacyl-tRNA (three substances), and arginine biosynthesis (three substances) pathways. Common differential metabolites enriched in the arginine pathway were also included in the amino acid and secondary metabolic biosynthesis pathways. The arginine biosynthesis pathway was the most significantly enriched pathway, according to the *p*-values. Among the 79 key differential metabolites, a total of three substances were enriched in the arginine biosynthesis pathway, namely, N-acetyl-ornithine, citrulline, and arginine. As shown in Figure 9, in the arginine synthesis pathway, N-acetyl-ornithine, citrulline, and arginine were significantly upregulated in all three group comparisons; glutamine, aspartate, putrescine (Put), and spermine (SPM) were significantly upregulated only in the first week; L-argininosuccinate was significantly upregulated while Put, spermidine (SPD), and SPM were significantly downregulated in the F2 vs. S2 and F3 vs. S3 groups; and N-acetyl-glutamate was significantly downregulated in the F2 vs. S2 group. We also performed a differential metabolite correlation analysis for 11 metabolites enriched in the arginine biosynthesis pathway (Figure 10). The results showed that N-acetyl-ornithine was negatively correlated with N-acetyl-glutamate, glutamate, and 2-oxoglutarate, while arginine was negatively correlated with N-acetyl-glutamate and 2-oxoglutarate. In addition, the other metabolites were positively correlated with each other.

## 3. Discussion

Hydroponics is a commonly used planting method in facility agriculture, and it is playing an increasingly important role in ensuring vegetable production and promoting botanical research development because of its advantages, such as a controllable growth environment, water conservation, soil-less cultivation, and environmental friendliness [1]. As previously mentioned, the cultivation substrate of hydroponics is a fluid, and this flowing nutrient solution can cause mechanical stimulation that affects root morphology and plant growth [5]. Studying the influence of nutrient solution flow on plant growth is important for understanding hydroponics. The root is a plant organ that comes into direct contact with the nutrient solution and plays an important role in thigmomorphogenesis caused by the nutrient solution flow. Considering that the formation of the root morphology is associated with metabolites [29,30,31], this study analyzed the roots of plants at different growth stages with and without flow conditions (F and S treatments, respectively), and the results showed that the yield of lettuce was reduced and the root became more compact under a high-flowing environment of nutrient solution.

The analysis results showed that the fresh weights of both shoots and roots in the F treatment were lower than those in the S treatment, although this difference was not significant in the early growth stage. The reason for this lack of significance may be that the metabolites affecting the phenotype require time to exert their influence. Although the metabolites of the roots grown under different flow conditions showed differential changes after approximately 7 d of treatment, it may take some time until these differences are translated into the phenotype. Additionally, we found that plants exhibited poorer growth when exposed to a high-flow environment, which is consistent with the conclusions of Baiyin et al. [4,5,6].

We also observed that certain parameters had higher values in the S treatment than the F treatment, such as the root total length, root surface area, and root volume, whereas higher values for root density and average root diameter were found in the F treatment. This suggests that a flow rate of 30 L/min may be excessive and result in thigmomorphogenesis. Thus, the roots adapt to the high-flow-rate environment by changing the morphology of their roots, which become shorter, thicker, and denser. Moreover, such changes in root morphology, especially the reduction in surface area, are unfavorable for nutrient absorption by plants, leading to decreased nutrient uptake and a slower growth rate.

We analyzed the composition of root metabolites under different flow conditions during different growth stages. An analysis of the PCA and OPLS-DA results showed that both the growth stage and flow treatment had an impact on metabolite composition. Even with the same treatment, the metabolites in the roots at different stages differed. This suggests that the concentrations and compositions of metabolites related to root morphology vary at different stages. From the OPLS-DA results, significant differences in metabolites were observed between the F and S treatments, regardless of the time period. Volcano plots comparing these three different growth stages showed that the number of upregulated metabolites was higher than the number of downregulated metabolites, indicating that the flow of nutrient solution may activate key physiological metabolic activities associated with changes in root morphology, leading to increased synthesis and regulation of certain metabolites under flow conditions. Identifying common differential metabolites with and without flow conditions during different growth stages may be crucial for explaining the mechanism of thigmomorphogenesis induced by nutrient solution flow.

Based on our analysis, we identified 79 common differential metabolites that are considered key metabolites influencing root thigmomorphogenesis. Cluster analysis of these 79 metabolites revealed that the F treatment had higher levels of 22 phenolic acids and 15 amino acids and their derivatives than the S treatment (Figure 7). Phenolic acids can act as antioxidants by helping plants counteract oxidative stress by neutralizing free radicals and mitigating cell damage caused by environmental stress [32]. Phenolic acids also play a role in cell division and elongation at the root apex, thus providing the necessary materials and energy for cell wall synthesis [33]. Amino acids and their derivatives have various effects on plant root growth and development, including protein synthesis, plant hormone activation, stress responses, and root morphology regulation [34]. These actions contribute to plant adaptations and normal root growth maintenance. Amino acids are fundamental proteins. During root growth, amino acids are involved in the synthesis of proteins required in the root apical cells [35], including enzymes and structural proteins. This is crucial for cell division, expansion, growth, and the extension of roots. Certain amino acids serve as precursors for plant hormones. For example, lysine can generate various hormones such as indole-3-acetic acid (IAA) and ethylene [36]. These hormones are involved in the regulation of root growth, root development, and lateral root formation. Certain amino acid metabolites play important roles in plant root stress responses. For example, glutamate and proline are important antioxidants that can neutralize free radicals and alleviate damage caused by environmental stress to the roots. Amino acids and their derivatives can also influence the morphology and spatial distribution of roots [37].

To identify the key metabolic pathways, KEGG enrichment analysis was performed on these 79 substances. These substances were mainly annotated and enriched in the arginine, amino acid, aminoacyl-tRNA, and secondary metabolite biosynthesis pathways (Figure 8). We analyzed the arginine biosynthetic pathway, which was the most significantly enriched metabolic pathway. The upstream and downstream metabolisms of arginine are shown in Figure 9. Among the 79 differentially expressed metabolites, three substances were enriched in the arginine biosynthesis pathway: N-acetylornithine, citrulline, and arginine. These three substances were significantly upregulated in all three comparison groups. Moreover, the metabolic network diagram revealed a positive correlation between these three metabolites.

Arginine plays a crucial regulatory role in plant root development. It promotes the formation and growth of root hairs and stimulates lateral root branching [38], regulates root development, and enhances root stress tolerance [39]. These functions contribute to the ability of plants to absorb water and nutrients, thereby enabling them to adapt to different environmental conditions. The specific mechanisms underlying arginine’s role in root development include regulatory effects exerted via its participation in nitric oxide signaling, polyamine synthesis, and hormone regulation pathways [40]. These mechanisms interact to collectively regulate root growth, differentiation, and stress responses, thereby influencing the absorption capacity, adaptability, and overall growth and development of plants.

Xia et al. [41] tested the effects of different concentrations of arginine on root elongation in both wild-type and mutant rice plants and noted that, in some plants, an appropriate concentration of arginine is essential for normal root growth. Wen et al. [42] found that the early addition of arginine significantly inhibited the growth of adventitious roots in *Tripterygium wilfordii*. However, different concentrations of arginine added at different time points had varying degrees of inhibitory effects on adventitious root growth. Glian`ko et al. [43] tested arginine concentrations of 0.1, 0.5, and 4 mM and found that 0.1 mM arginine had no effect while 4 mM arginine negatively affected root growth. The inhibitory effect of high doses of arginine on growth may be attributed to the accumulation of other compounds synthesized in conjunction with nitric oxide, such as peroxynitrite [44].

Arginine serves as a precursor for the generation of nitric oxide (NO) and polyamines (PAs), and it participates in multiple physiological and pathological processes in both animals and plants by transforming into signaling molecules such as PA and NO [45]. NO is an extremely unstable biological free radical present in various tissues. It has broad physiological effects and acts as a messenger in both animals and plants [46]. Auxins can regulate root growth and development by activating protein kinases and can induce the formation of adventitious roots via NO. Additionally, NO activates MAPK and acts as an intermediate messenger in auxin-induced lateral root initiation [47,48]. Gouvea et al. [49] observed that NO is involved in promoting root growth and found that NO induces cell elongation in a manner similar to that induced by auxins.

In addition to being catalyzed by nitric oxide synthase (NOS) to form NO, arginine can also be converted into agmatine via the action of arginine decarboxylase (ADC) and then further hydrolyzed by agmatinase to form PAs. It can also be converted into ornithine by the action of arginase, and under the catalysis of ornithine decarboxylase (ODC), ornithine is further transformed into PAs, which can then be further converted into other PAs, such as Put, SPD, and SPM [50,51,52]. PAs are a class of low-molecular-weight aliphatic nitrogenous bases that exhibit physiological activities within organisms [53]. They are involved in various physiological and biochemical processes and reactions, such as cell division, DNA condensation, membrane stabilization, hormone signal transduction, and stress resistance, in both animals and plants [54].

Exogenous PAs and arginine have been shown to increase the number of new roots in apple seedlings [55]. PA promotes root growth at low concentrations but exerts inhibitory effects at high concentrations [56]. This effect of PA has been attributed to the regulation of cell division [57]. In summary, the regulatory effects of arginine and its metabolic products on root development may be similar to those of auxin. For example, it promotes growth in small quantities but inhibits growth in larger quantities. It is speculated that, in comparison to the S group, in the F group, the enhanced regulation by arginine and hormones involving arginine may lead to excessive promotion, resulting in the inhibition of root elongation and growth, leading to a shorter, thicker, and denser root morphology.

This study did not directly test the effects of NO or provide or consider explanations at the RNA, hormonal, or protein levels. To gain a more comprehensive and accurate understanding of the study results, future research should directly detect and measure the levels and activity of NO in cells using appropriate experimental techniques, such as chemical probes or biochemical methods, to verify the impact of arginine on NO production. Transcriptomic analysis should also be conducted to study the changes in gene expression related to arginine in cells after flow treatment. Such work will provide a better understanding of the regulatory role of flow in arginine-related gene transcription levels and its potential interactions with gene signaling pathways associated with NO production. Additionally, further investigations should be performed on the effects of arginine on the levels of hormones, particularly those related to NO and plant growth, such as the regulation of NOS activity, NO release, and auxin synthesis. Furthermore, this study focused on identifying the key metabolites that influence thigmomorphogenesis and provided a preliminary exploration of the effects of nutrient solution flow on thigmomorphogenesis. Only the key metabolites and search processes are explained. In addition, the methodology used in this study took advantage of stable environmental conditions and ease of handling; however, it is not perfect because the dissolved oxygen of nutrient solutions in the S and F treatments might be different, and the results might be affected. Therefore, gradient designs for flow treatments will be conducted in the future to determine the differences in plant growth and metabolites under different flow rates, which will further validate and expand upon the conclusions of this study. Such work could be achieved via an appropriate experimental design and biochemical analysis. By further analyzing these factors, more comprehensive research results can be obtained, which will provide insights into the mechanisms underlying the effects of arginine, including its interaction with NO, transcription factors, hormones, and proteins. Further studies will help reveal the effects of arginine on thigmomorphogenesis and its interactions with NO at the transcriptional, hormonal, and protein levels.

## 4. Materials and Methods

### 4.1. Cultivation

The plant used in this study was lettuce (*Lactuca sativa* L.) (long-tillage; Hebei Manchou Agri-Tech Co., Ltd., Shijiazhuang, China). First, the seeds were placed in seedling trays filled with moist vermiculite for germination. Seven days after sowing, seedlings with consistent growth were selected and transplanted into plastic containers containing 20 L of a standard nutrient solution for seven days of cultivation. Then, seedlings with relatively consistent growth were planted in cultivation tanks (length: 1.7 m, width: 0.2 m, height: 0.2 m). Each cultivation tank was filled with 50 L of standard nutrient solution (pH = 5.7 and EC = 2.25). Each treatment (F1–F3, S1–S3) had three replicates (three cultivation tanks), with five plants per cultivation tank. A commercially available nutrient solution (vegetable and flower general nutrient solution; Caijudongli Agri-Tech Co., Ltd., Zhengzhou, China) was used for the hydroponic experiment. The compositions and concentrations of the standard nutrient solutions are listed in Table 1.

**Figure 11:**
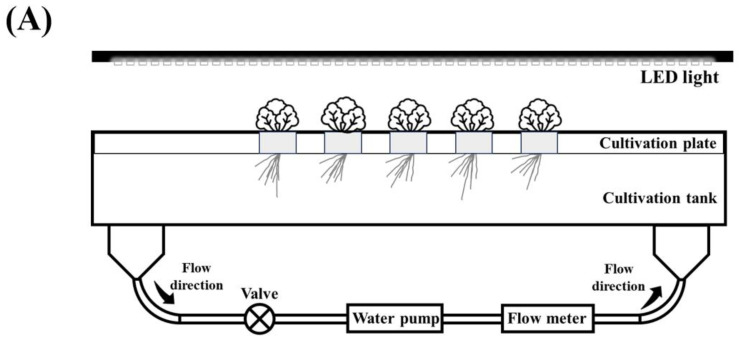

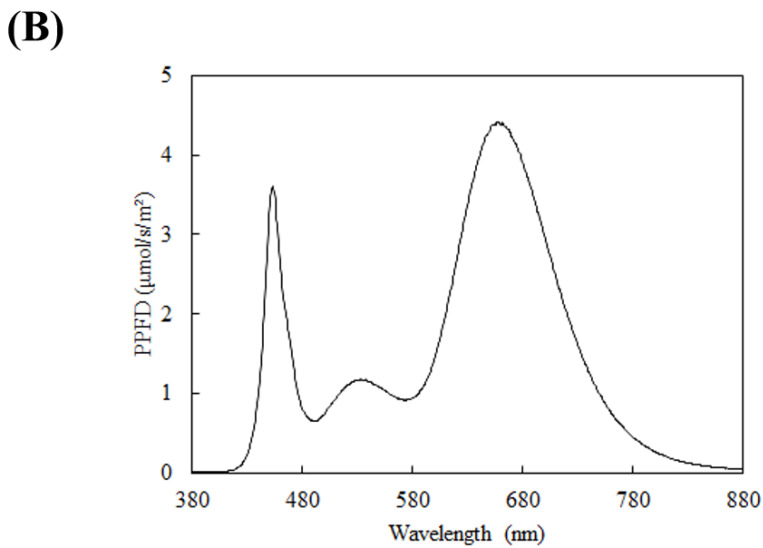
Schematic diagram of the cultivation device (**A**) and spectrum information of the LED light used in this study (**B**).

The diagram of the cultivation device used in this study is shown in Figure 11A. The nutrient solution was circulated using a water pump (XDP-7500, SENSEN GROUP Co., Ltd., Zhoushan, China), and the flow rate of the nutrient solution was monitored using a digital flow meter (display: ZJ-LCD-S2, and sensor: B7; Zhongjiang Dianzi Co., Ltd., Shenzhen, China). LED lights (WZ-90, Shandong Guixiang Guangdian Co., Ltd., Weifang, China) were used for illumination during the experiment. Light intensity on the cultivation plate was measured using a light meter (AvaSpec-ULS2048XL-RS-EVO, Avantes, the Netherlands). The photosynthetic photon flux density (PPFD) was approximately 550 µmol/s/m^2^ (spectrum information in Figure 11B). The light cycle was set to 16 h:8 h (light/dark). The average ambient temperature of the cultivation room during the cultivation period was 25 ± 1 °C, and the relative humidity was 65 ± 5%.

### 4.2. Measurement and Statistical Analysis

#### 4.2.1. Fresh Weight and Root Morphology

Sampling was conducted 1–3 weeks after planting, and the shoot and root samples were separated. First, an electronic balance (FA2204, Shanghai Lichen Co., Ltd., Shanghai, China) was used to measure the plant’s fresh weight. A root scanning system and analysis software (WinRhizo 2013, Regent INS, Quebec, QC, Canada) were used to measure the total root length, root surface area, root volume, and average root diameter. For samples intended for metabolite analysis, the root samples were immediately flash-frozen in liquid nitrogen for 15 min after sampling and then stored at −80 °C.

#### 4.2.2. Metabolite Extraction and Detection

The plants were harvested at different time points in both conditions for widely targeted metabolite analysis. Metabolite extraction was performed according to the protocol described by Zhang et al. [58]. First, the entire root of a lettuce plant was placed in a freeze dryer (Scientz-100F, Scientz, Ningbo, China) for vacuum freeze-drying, followed by grinding using a grinder (MM 400, Retsch, Germany) at 30 Hz for 1.5 min until a powder form was obtained, which was then thoroughly mixed. Next, 50 mg of freeze-dried powder was accurately weighed and added to 1200 μL of −20 °C pre-chilled 70% methanol–water internal standard extraction solution. The mixture was vortexed six times (every 30 min, with each vortex lasting 30 s). After centrifugation at 12,000 rpm (11,304× *g*) for 3 min, the supernatant was collected and filtered through a microporous membrane (0.22 μm pore size) and then stored in sample vials for UPLC-MS/MS analysis. QC samples were prepared by mixing equal amounts of supernatants from all samples.

Then, an UPLC-ESI-MS/MS system (UPLC, ExionLC AD, https://sciex.com.cn/ (accessed on 2 September 2023); tMS/MS, Applied Biosystems 6500 QTRAP, http://www.appliedbiosystems.com.cn/ (accessed on 2 September 2023), Framingham, MA, USA) was used to analyze the root extracts. The analytical conditions were as follows [59]: UPLC: column, Agilent SB-C18 (1.8 µm, 2.1 mm × 100 mm); mobile phase: solvent A, pure water with 0.1% formic acid; solvent B, acetonitrile with 0.1% formic acid. Sample measurements were performed using a gradient program that employed starting conditions of 95% A and 5% B. Within 9 min, a linear gradient of 5% A and 95% B was programmed, and a composition of 5% A and 95% B was maintained for 1 min. Subsequently, the composition was adjusted to 95% A and 5.0% B within 1.1 min and maintained for 2.9 min. The flow velocity was set at 0.35 mL per minute; the column oven was set to 40 °C; and the injection volume was 2 μL. The effluent was alternately connected to an ESI-triple quadrupole linear ion trap (QTRAP)-MS.

The ESI source operation parameters were as follows [60]: source temperature, 500 °C; ion spray voltage (IS), 5500 V (positive ion mode)/−4500 V (negative ion mode); ion source gas I (GSI), gas II (GSII), and curtain gas (CUR), 50, 60, and 25 psi, respectively; the collision-activated dissociation (CAD) was high. QQQ scans were acquired in the multiple reaction monitoring (MRM) experiments with a collision gas (nitrogen) in the medium. The declustering potential (DP) and collision energy (CE) for individual MRM transitions were determined via further DP and CE optimization. A specific set of MRM transitions was monitored for each period according to the metabolites eluted within this period [61].

#### 4.2.3. Data and Statistical Analysis

Based on the MWDB database (Metware Biotechnology Co., Ltd., Wuhan, China) and public databases, the substances were qualitatively analyzed based on information from the MS/MS spectra. The metabolites were quantitatively analyzed using the MRM [62]. Analyst software (version 1.6.3, Sciex, Framingham, MA, USA) was used to process the mass spectrometry data.

The PCA was performed using the built-in statistical function prcomp in R software (www.r-project.org/ (accessed on 5 September 2023)). Metabolite abundance data were preprocessed using unit variance scaling (UV), and the accumulation patterns of metabolites among different samples were determined via hierarchical cluster analysis (HCA). The ComplexHeatmap package in R software was utilized to create a clustering heatmap. Furthermore, the built-in cor function in R was used to calculate Pearson’s correlation coefficient (r) to evaluate the correlations among biological replicates.

The OPLS-DA was used to conduct log2 conversions on the raw data and then for performing centralized processing. The MetaboAnalystR package OPLSR’s anal functions in R software were then used for analysis [63]. Score maps were drawn for each group, and VIP (variable importance in projection) values were obtained to preliminary screen for intergroup differential metabolites (VIP > 1). The FC (Fold change) univariate statistical analysis method was applied for further screening of differential metabolites (FC ≥ 2.0 or FC ≤ 0.5).

The KEGG Compound database (http://www.kegg.jp/kegg/compound/ (accessed on 9 October 2023)) was used to annotate the identified metabolites, and then the annotated metabolites were mapped to the KEGG Pathway database (http://www.kegg.jp/kegg/pathway.html (accessed on 9 October 2023)). Pathways mapped to significantly regulated metabolites were then assessed via metabolite set enrichment analysis (MSEA), and their significance was determined using hypergeometric test *p*-values [58].

SPSS (version 26, IBM, Chicago, IL, USA) was used for the statistical analysis of the plant growth indicators. All data were statistically analyzed using independent sample *t*-tests, and the statistical results were expressed as the mean ± standard error (*n* = 5). A value of *p* ≤ 0.05 indicated significant differences.

## 5. Conclusions

The growth of lettuce was inhibited by a high-flowing environment of nutrient solution, resulting in a lower yield and a more compact root. By comparing the accumulation of differential metabolites, we found that the root of lettuce produced more metabolites, such as amino acids and their derivatives, as well as phenolic compounds, under a high-flow environment. These metabolites may be associated with plant defense responses and adaptability. Moreover, arginine biosynthesis was considered a key pathway that influenced the root development of lettuce. The findings would help understand how root thigmomorphogenesis is induced by a high-flowing environment of nutrient solution in hydroponics from the perspective of metabolism.

## Figures and Tables

**Table 1:** The composition and concentration of the standard nutrient solution.

Composition	Concentration
Ca(NO₃)₂·4H_2_O	945 mg/L
KNO₃	607 mg/L
NH₄H₂PO₄	115 mg/L
MgSO₄·7H₂O	493 mg/L
Na_2_Fe(EDTA)	20–40 mg/L
H₃BO₃	2.86 mg/L
MnSO_4_·4H₂O	2.13 mg/L
ZnSO₄·7H₂O	0.22 mg/L
CuSO₄·5H₂O	0.08 mg/L
(NH_4_)_6_Mo_7_O_24_·4H_2_O	0.02 mg/L

## Data Availability

All data generated or analyzed during this study are included in this published article.
